# Understanding Emotional Labor Dynamics in Participant Sport Service: A Conceptual Framework

**DOI:** 10.3390/bs13090771

**Published:** 2023-09-15

**Authors:** Ye Hoon Lee

**Affiliations:** Division of Global Sport Industry, Hankuk University of Foreign Studies, Seoul 17035, Gyeonggi-do, Republic of Korea; leeye22@hufs.ac.kr; Tel.: +82-31-330-4986

**Keywords:** emotions, emotional regulation, participant sport, service management, well-being, customer outcomes

## Abstract

In the service industry, the quality of interactions between employees and customers is crucial for improving customer outcomes. Emotional labor, which involves managing one’s emotions to meet job requirements, is a significant aspect of these interactions. To address the lack of attention paid to emotional labor in participant sport contexts, this study proposes a conceptual framework that outlines the antecedents, consequences, and moderators of emotional labor strategies in participant sport service. The framework includes 25 propositions based on theories and empirical evidence from various scientific domains. More specifically, the conceptual framework consists of four main components: (a) intrapersonal and (b) interpersonal consequences; (c) moderators; and (d) antecedents of emotional labor. The study recommends that sport organizations should implement emotional labor training workshops to help employees engage in effective and health-beneficial emotional labor strategies specific to sport organization settings. Overall, this conceptual framework provides a foundation for understanding emotional labor in sport organizations and can help enhance customer outcomes and employee well-being in the participant sport service industry.

## 1. Introduction

Based on the report by Statista [1], the global sports industry had a market value of USD 487.6 billion in 2022, and it is expected to grow at a Compound Annual Growth Rate (CAGR) of 5.2%, reaching USD 623.63 billion by 2027. Sponsorship is identified as the main source of revenue in sports, with USD 55.3 billion generated in 2022, followed by media rights at USD 34.2 billion and ticket sales at USD 22.1 billion. Significant sports events like the Olympics and World Cup also generate substantial revenue, with the 2016 Summer Olympics earning USD 5.8 billion according to the International Olympic Committee (IOC). Furthermore, the National Football League (NFL) recently signed a 10-year media rights agreement worth USD 113 billion with various broadcasters in March 2021, marking the most substantial media rights deal in the history of sports [2].

While these optimistic figures mostly represent the size of “spectator” sports, which have received the most attention from sport researchers [3], it is the “participant” sport sector that makes up a larger share of the industry [4]. For example, 19.3% of Americans participated in sports and exercise every day in 2019. Over 60 million people ran, jogged, or participated in trail running in 2019, compared to over 50 million people who hiked. Notably, among the generations, Gen Z (born in 2000 or later) reported participating in outdoor activities at the greatest percentage (59.1%) in 2019 [5]. As people have more free time and become more concerned with issues like obesity and healthy aging, they tend to engage in more sports activities. However, as this industry continues to gain popularity, competition between organizations has become a major concern [6]. As a result, participant sport organizations make efforts to set themselves apart from their rivals to keep their current customers satisfied and to entice new ones to join [7,8]. To achieve their goals and survive in a highly competitive industry, these organizations attempt to provide high-quality services that have the potential to impact customer satisfaction, which, in turn, helps them build long-term relationships with their customers [9].

Human capital plays a crucial role in developing a competitive advantage in participant sport contexts because the attitudes and behaviors of employees during direct interactions with customers influence their perceptions of the service received [10,11]. In the participant sport industry, service employees who have the ability to provide excellent service in the customer–employee interface play a crucial role in establishing a competitive advantage and determining the success of sport organizations. As part of their job responsibilities, service employees are expected to engage in emotional labor, a term referring to the intentional effort to express specific emotions required by the organization while suppressing others, during their direct interactions with customers [12]. Many studies have shown that emotional labor is significantly associated with the individual well-being of employees, including job satisfaction and job burnout (please see a meta-analysis of Hulsheger and Schewe [13]), as well as with organizational outcomes, such as perceived service quality and customer satisfaction [14,15] in various job settings, including tourism [16], the fitness industry [17], and education [18], and among athletic coaches [19], professors [20], and government employees [21]. The significance of emotional labor in service sectors is well recognized [22], but research on its connection to participant sports is limited. Participant sports involve unique features of service provision, and service providers must make conscious efforts to develop positive employee–customer interfaces to ensure a pleasurable experience for the customer [11]. Therefore, it is crucial to explore the function of emotional labor in these customer–employee interactions [17].

The aim of this study is to establish a conceptual framework for understanding the importance of emotional labor in the sports industry. Previous research on emotional labor in various domains was analyzed, and its potential application in sport participation organizations was discussed. The objective is to improve our understanding of the emotional challenges that participant sport service employees may face, the effects of emotional labor on customers, and the approaches that employees adopt to regulate their emotions. This knowledge can assist in the development of training programs and marketing systems for participant sport service employees and contribute to the broader discourse on the role of emotions in sports.

## 2. Theoretical Framework

### 2.1. Emotional Labor and Emotional Labor Strategies

The concept of emotional labor, as defined by Grandey [23], involves the regulation of both emotional experiences and their expressions in order to conform to the display rules set by organizations to achieve desired outcomes. This means that employees must manage their emotions and expressions to meet the standards defined by their employers. For instance, a personal trainer may need to display enthusiasm and encouragement even when feeling tired or frustrated while working with a client struggling with an exercise. Display rules are guidelines that determine which emotions employees should show and suppress to be effective in their work [23]. Display rules, on the other hand, define appropriate emotional expressions in relationships and roles, and they guide emotional conversations. Emotional labor, therefore, entails acts of emotion management that occur in social exchanges guided by feeling rules [12].

Grandey [23] classified display rules into three distinct types, namely integrative, differentiating, and masking. The integrative display rule requires employees to exhibit positive emotions and develop warm relationships with customers. For instance, customer service representatives must display cheerfulness when interacting with customers. On the other hand, differentiating display rules entail displaying negative emotions that drive people apart, such as anger, fear, or hate. An example of this is debt collectors who may need to display negative emotions like aggressiveness and anger when pursuing borrowers for payment. Finally, masking emotions require employees to maintain neutrality during interactions with others.

Scholars have classified emotional labor strategies into three categories [12,23,24]. The first strategy is surface acting, where employees modify their outward emotional expressions to comply with display rules without changing their inner emotions. The second strategy is deep acting, where employees make a genuine attempt to feel the required emotion as prescribed by the display rules. Deep acting is considered more authentic than surface acting as it involves a sincere effort to modify one’s internal emotional state.

Ashforth and Humphrey [24] proposed the expression of genuine emotion as the third emotional labor strategy, which involves employees naturally and authentically experiencing and exhibiting appropriate emotions without relying on surface or deep acting. For instance, athletic coaches may feel genuinely passionate about sports, and therefore, may not need to deliberately engage in surface or deep acting. Nevertheless, this expression is still considered emotional labor because employees are required to perform the emotions necessary for the organization. Although Grandey and Gabriel [22] raised doubts about whether genuine expression should be classified as an emotional labor strategy, most researchers contend that even though it may occur involuntarily, genuine expression requires a change in inner feelings and should be regarded as a form of emotional labor [25]. For example, when tennis players execute a forehand stroke, they do so automatically based on previous practice, without any conscious effort. Despite not requiring deliberate cognitive or physical processes during competition, tennis players are still performing physical labor or work. Similarly, even though genuine expression may occur automatically, as it aims to achieve organizational goals, it should be included as one of the emotional labor strategies. A study conducted by Diefendorff, Croyle, and Gosserand [26] demonstrated that genuine expression of emotions is considered a distinct approach to managing emotions in the workplace.

### 2.2. Theoretical Background of Intrapersonal and Interpersonal Effects of Emotional Labor

#### 2.2.1. Intrapersonal Mechanism

Prior studies on emotional labor have mainly concentrated on the effects of emotional labor within individuals. This study explores how the utilization of different emotional labor techniques affects an individual’s cognition, behavior, and overall well-being. The section introduces theories that elucidate the psychological processes involved in this intrapersonal process, specifically Conservation of Resource Theory (COR) [27] and Psychological Cost [28].

The Conservation of Resource (COR) theory, proposed by Hobfoll [27], offers an explanation of how emotional labor strategies can impact an individual’s well-being, such as job burnout and work attitude such as job satisfaction. The theory posits that people aim to safeguard, maintain, and accumulate resources such as possessions, personal traits, circumstances, and energy. When individuals perceive a decline or danger to these resources, stress can occur. They cope with this stress by using strategies such as replacement, coping, and appraisal. COR theory posits that individuals experience negative consequences (e.g., job burnout) when the investment of their resources leads to a lower future return and experience positive consequences (e.g., job satisfaction) when the investment leads to a net gain in resources.

However, emotional labor is a coping mechanism that depletes an individual’s inner resources since it requires conscious regulation and self-control [23]. Thus, when individuals face stressful situations such as challenging customers, they tend to use emotional resources to cope with the situation, hoping to obtain positive results. This mechanism is called emotional investment. Brotheridge and Lee [29] suggest that if the investment is successful, and individuals receive a net gain of resources, they may experience eustress. However, if the returns on investment are perceived as inadequate compared to the amount of emotional resources expended, individuals may experience negative outcomes [20].

Lee and colleagues [28] note that emotional labor strategies can have negative impacts on individual outcomes, including job-related attitudes (e.g., job satisfaction and organizational commitment), overall health, and well-being (e.g., job burnout and stress) due to the psychological cost. The first reason for this is that some strategies may result in emotional dissonance, where there is a discrepancy between the emotions a person experiences and those they are required to display. This can cause psychological discomfort, leading to physical and mental harm. Secondly, certain strategies require significant psychological effort, as individuals must monitor their behaviors to ensure their emotional display aligns with situational requirements, leading to cognitive strain [4]. Finally, emotional labor can also generate a sense of insincerity, resulting in depersonalization and a sense of estrangement from one’s job. Different dimensions of emotional labor can result in various degrees of these psychological processes, which can ultimately influence employees’ job-related well-being. Research shows that emotional dissonance is significantly associated with negative job attitudes and impaired well-being [13,30].

#### 2.2.2. Interpersonal Mechanism

While most studies on emotional labor have focused on its personal impact, there is a need to understand its social nature as well [22]. As emotions can be observed and potentially influence others’ behavior, it is crucial to examine the interpersonal aspects of emotional labor. To address this, the following section will introduce Emotional Contagion Theory [31], which provides insight into the interpersonal mechanisms involved in emotional labor.

The theory of emotional contagion can explain how emotional labor affects customers’ perceptions and organizational outcomes. Emotional contagion refers to the phenomenon where an individual automatically imitates the facial expressions, vocalizations, postures, and movements of another person, resulting in a convergence of emotions between them [31]. This process can be passed on from one person to another through mirror-neuron activity, mimicry, and afferent feedback. Studies have shown that emotional contagion is a well-supported phenomenon in service contexts, where shared emotions between customers and clients impact service quality judgments (e.g., [32,33,34]). Emotional contagion can also occur in sports teams, where athletes’ emotions can influence their teammates’ emotional states and subsequently affect their individual performances. For example, Tamiminen and Crocker [35] found that athletes’ non-intentional emotional displays can influence the emotional states of their teammates. In the context of participant sports, it is possible that personal trainers who express their emotions with passion may lead members to feel that same emotion, resulting in positive affective reactions and subsequent revisits to the participant sport organizations. Conversely, tennis instructors who display irritation may negatively impact members’ emotional states, resulting in a loss of motivation and ultimately quitting their lessons. By understanding how emotional labor and emotional contagion can impact customer behavior, sports organizations can develop strategies to enhance customer satisfaction and retention.

### 2.3. Emotional Labor in Participant Sport Service Contexts

According to Chelladurai [11], participant sport services refer to services that facilitate clients’ participation in sports or physical activities. Individuals who participate in sports programs, such as swimming lessons, personal training sessions, and youth sports leagues, with the aim of enhancing their athletic skills, fitness levels, or recovering from injuries, are considered participants within sport organizations. Service providers in these settings may encounter instances of customer incivility, which can manifest as rudeness or excessive demands. For example, participants might express frustration or rudeness when they perceive that the facilities, equipment, or amenities provided by the sport organization do not meet their expectations. Instructors or trainers may also encounter participants who repeatedly ignore safety rules or disrupt the flow of a class, requiring them to address these issues tactfully. Further, in youth sports leagues, parents may become overly demanding, constantly questioning coaching decisions, or even displaying rude behavior during games, which can create a tense atmosphere. Consequently, the ability to manage emotions becomes pivotal for effectively handling such negative incidents, benefiting both employees and organizations in the fulfillment of their roles [23].

Within this context, emotional labor can be crucial for creating a positive and supportive environment that encourages individuals to achieve their goals. For example, personal trainers or instructors must not only arrange lessons and transmit technical knowledge for individualized training programs; they must also demonstrate emotional intelligence and social skill to motivate and support their members [36]. This complicated function demands emotional work, which includes the skill of controlling one’s emotions to project positivity and enthusiasm. It also requires the ability to tune into the emotional states of the clients and respond appropriately to their specific requirements. For instance, in order to help a member overcome emotional obstacles that stand in the way of achieving their fitness goals, a personal trainer may find it essential to use empathy and active listening techniques.

Secondly, the rise of technology has made it easier for people to work out at home, which has led to a decline in participant sport attendance [37,38]. In this context, emotional labor is even more important for participant sport instructors in such areas as participant sport organizations, as they compete with the convenience of home workouts. When people choose to work out at home, they are often seeking a more personalized and convenient experience. However, this personalized experience can also come at the cost of accountability, motivation, and social support. This is where participant sport instructors can differentiate themselves by utilizing emotional labor to create a supportive and motivating environment.

By utilizing emotional labor, participant sport instructors can build relationships with their clients, provide personalized attention, and motivate them to achieve their goals. For example, an instructor can use positive reinforcement, provide social support, and use active listening skills to understand and respond to the emotional needs of their clients [36]. This can lead to a more effective workout experience and greater adherence to goals. Moreover, when participant sport instructors are skilled in utilizing emotional labor, they can create a sense of community within the participant sport organizations. This can help to counteract the isolation and lack of accountability that can come with home workouts. By building a sense of belonging and connection, participant sport instructors can make it more likely that clients will continue to come to the gym for their workouts.

Thirdly, participant sport contexts should focus on emotional labor not only for financial reasons, but also to contribute to society’s well-being. Physical activity greatly benefits individuals’ mental and physical health while physical inactivity is a leading cause of death [39]. Therefore, participant sport organizations should integrate physical activity promotion to encourage more people to be active more frequently. One effective way to achieve this is by improving the retention rate of participant sport organizations. Emotional labor is a critical factor in maintaining participants’ engagement in sport activities. When coaches and staff can connect with participants emotionally, they are more likely to continue participating in sports activities and recommend the organization to others. This can ultimately lead to higher retention rates and greater financial success for the organization. Studies have shown that emotional labor impacts customer loyalty intention [14,40], making it an effective tool for increasing engagement and retention in sports organizations.

## 3. Conceptual Framework

**Figure 1:**
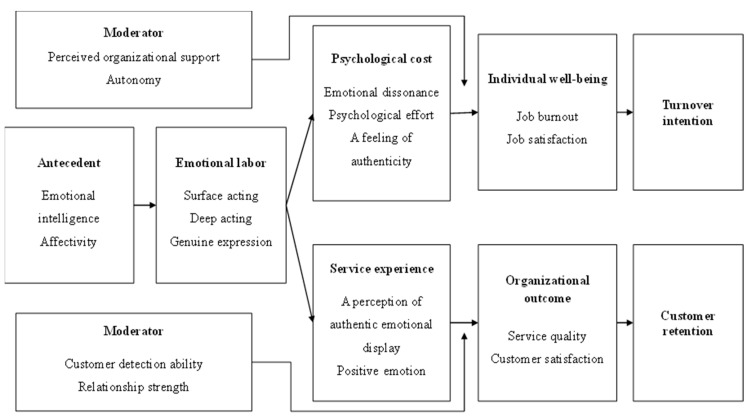
A conceptual framework of emotional labor in participant sport service.

This conceptual framework (Figure 1) consists of four main components: (a) intrapersonal and (b) interpersonal consequences ; (c) moderators; and (d) antecedents of emotional labor.

### 3.1. Emotional Labor and Occupational Well-Being

Hochschild [12] argued that emotional labor could lead to negative outcomes such as job burnout, low job satisfaction, and psychological distress. Recent meta-analyses [13,41,42,43] have provided evidence to support this argument.

#### 3.1.1. Emotional Labor and Job Burnout

Job burnout is a commonly observed result of emotional labor. According to Maslach and Jackson’s [44] definition, job burnout refers to the occurrence of emotional exhaustion, depersonalization, and reduced personal accomplishment among individuals who engage in work that involves interacting with people in some capacity. Hochschild [12] argued that emotional laborers were particularly vulnerable to burnout due to the direct interaction between employees and customers. Numerous studies have supported this relationship (for a review, see [13]). However, Johnson and Spector [45] have suggested that using different emotional labor strategies can result in different consequences.

Surface acting, which involves high levels of all three types of psychological cost, has been found to be a health-detrimental strategy. It creates a discrepancy between employees’ internal feelings and their outward expressions, leading to high levels of emotional dissonance and a feeling of inauthenticity. Additionally, surface acting requires employees to exert significant psychological effort by suppressing their internal feelings, faking their outward expressions, and monitoring their behaviors to ensure that their expressions are aligned with situational requirements [28].

On the other hand, research suggests that deep acting and genuine expression can have positive effects on individual well-being. Deep acting, which incurs moderate psychological costs, and genuine expression, which does not require any costs, have been linked to reduced job burnout [13,19,46,47,48]. Deep acting involves adjusting one’s perception of the situation to align with inner emotions, resulting in a match between felt and displayed emotions. In contrast to surface acting, deep acting requires some degree of psychological effort but is associated with lower emotional dissonance and a lesser feeling of inauthenticity. Furthermore, genuine expression involves a natural alignment of felt emotions with expressed emotions, resulting in minimal emotional dissonance or inauthenticity. Moreover, this strategy does not require psychological effort as it is an automatic process [28]. Based on the above, it is proposed that the following propositions hold true.

**Proposition** **1.** *Surface acting is expected to increase job burnout*.

**Proposition** **2.** *Deep acting is expected to decrease job burnout*.

**Proposition** **3.** *Genuine expression is expected to decrease job burnout*.

#### 3.1.2. Emotional Labor and Job Satisfaction

Emotional labor has also been linked to job satisfaction, which refers to how much individuals like or dislike their jobs [47,48,49,50]. The relationship between emotional labor strategies and job satisfaction can be explained by the level of psychological cost involved [28]. For instance, surface acting, which requires significant psychological effort and results in a feeling of inauthenticity and emotional dissonance, is associated with decreased job satisfaction. Cheung and Lun [47] suggested that employees who suppress their true emotions and display fake emotions experience a high level of inauthenticity, which, in turn, leads to job dissatisfaction and the intention to quit. In contrast to surface acting, deep acting has been found to have a positive relationship with job satisfaction. Deep acting enables employees to feel authentic by matching their inner emotions with their displayed emotions, which is well received by customers in transactional contexts. This positive feedback helps employees replenish their inner resources and overcome the emotional effort involved, resulting in higher job satisfaction [28]. This is supported by studies such as Cheung and Lun [47], Lee and Chelladurai [19], Li and Wang [48], and Yin [49]. Finally, genuine expression generates no emotional dissonance, psychological effort, or feelings of inauthenticity, and may enhance job satisfaction among employees through resource gains due to positive social feedback [28].

**Proposition** **4.** *Surface acting is expected to decrease job satisfaction*.

**Proposition** **5.** *Deep acting is expected to increase job satisfaction*.

**Proposition** **6.** *Genuine expression is expected to increase job satisfaction*.

#### 3.1.3. Job Burnout, Job Satisfaction, and Turnover Intention

Turnover intention is when an employee wants to leave their job or organization, and it is a significant concern for service-based organizations due to the negative impact it can have on the business and the high cost of losing valuable employees. Previous studies in different contexts have repeatedly demonstrated a positive link between job burnout and turnover intention [51,52,53], which eventually affects employees’ actual withdrawal behavior [54]. The Conservation of Resources (COR) theory explains this association by proposing that individuals aim to maximize their net gains and compensate for the net loss of resources through strategies such as replacement when they experience an actual resource depletion. Job burnout syndrome indicates a loss of one’s internal energy [44], which can be perceived as a loss of resources among individuals. To compensate for this loss and maximize net gains, employees may consider withdrawing from their current job and looking for opportunities to work in other occupations or organizations. Several studies have demonstrated that job satisfaction is negatively associated with employee turnover intention [19]. A meta-analysis conducted by Li and Yao [55] also found a significant and negative correlation between job satisfaction and turnover intention.

**Proposition** **7.** *Job burnout is expected to increase turnover intention*.

### 3.2. Moderator of Intrapersonal Consequence

To mitigate the adverse effects of emotional labor strategies on employee well-being outcomes, it is crucial to identify moderators that can reduce the negative implications of surface acting. Through a literature review, three potential moderators were identified that can be applied to participant sport services.

First, perceived organizational support refers to employees’ perceptions of how much their organization values and cares about them and their contributions [56]. Employees attribute human-like qualities to their organization, which can range from supportive to malevolent, shaping their overall beliefs about the organization. Perceived organizational support plays a moderating role in the relationship between emotional labor and employee well-being, which can be explained through the principles of COR theory [27], including the norm of reciprocity. As mentioned earlier, COR theory suggests that individuals experience stress and negative outcomes when their resources, such as physical, cognitive, or personal resources, are depleted. Support from organizations can act as a resource supplement, replenishing employees’ inner resources and mitigating the negative impact of surface acting, while also enhancing the positive impact of genuine expression.

**Proposition** **8.** *Employees with high perceived organizational support are expected to have a weaker positive relationship between surface acting and job burnout and a stronger negative relationship between surface acting and job satisfaction compared to employees with low perceived organizational support*.

**Proposition** **9.** *Employees with high perceived organizational support are expected to have a stronger negative relationship between genuine expression and job burnout and a stronger positive relationship between deep acting and job satisfaction compared to employees with low perceived organizational support*.

Secondly, autonomy refers to an individual’s active participation in determining their own behavior [57]. In the context of emotional labor, autonomy is reflected in whether an individual feels the need to adhere to organizational emotional display rules (i.e., engage in emotional labor [58]). Previous literature has noted that an employee’s feeling of autonomy can have a significant impact on the emotional labor–individual outcome relationship [45]. The demands–control model by Karasek and Theorell [59] suggests that high job demands (such as display rules) can be more stressful for employees with low autonomy over their tasks and behaviors at work. This highlights the importance of control in the stressor–strain relationship, where employees with high job demands and control tend to perceive negative events as a challenge rather than a strain. Thus, employees who perceive that they have autonomy in expressing their own emotions will experience fewer negative consequences, while those who feel they have less latitude in expressing their emotions will experience more negative consequences.

**Proposition** **10.** *The association between surface acting and individual well-being may be influenced by job autonomy*.

**Proposition** **11.** *The association between genuine expression, deep acting, and individual well-being may be influenced by job autonomy*.

### 3.3. Emotional Labor and Organizational Outcomes

As mentioned earlier, while emotional labor may have negative effects on individual well-being of employees in participant sport organizations, it can also have positive effects on organizational outcomes [22,60]. This model includes perceived service quality and customer satisfaction as variables, which in turn influence customer loyalty intentions.

#### 3.3.1. Perceived Emotional Labor and Customer Satisfaction

Customer satisfaction refers to the combination of cognitive and emotional responses to a specific service encounter [61]. As it has a significant influence on an organization’s profitability, marketing directors prioritize it [62,63]. Satisfied customers are more likely to re-use or repurchase services, including those offered by sport organizations [7,64,65].

Studies have revealed that emotional labor resulting in inauthentic displays can have a negative impact on customer service experiences and evaluations of employees [14,44,66]. Inauthentic displays can lead to unfavorable judgments about the employee’s honesty, pleasantness, and likability [67,68], as well as trust and cooperation [69]. The authenticity of emotional displays resulting from employee emotional labor can affect service evaluations. Empirical research indicates that when employees engage in deep acting and genuine expression, customers are more likely to be satisfied with the interaction [13,15]. Conversely, surface acting, which involves suppressing negative emotions and faking outward expression, has a negative relationship with customer satisfaction [42].

**Proposition** **12.** *There is expected to be a negative relationship between surface acting and customer satisfaction*.

**Proposition** **13.** *There is expected to be a positive relationship between deep acting and customer satisfaction*.

**Proposition** **14.** *There is expected to be a positive relationship between genuine expression and customer satisfaction*.

#### 3.3.2. Perceived Emotional Labor and Perceived Service Quality

Perceived service quality is defined as the difference between a customer’s expectations and their actual experience of the service they receive [63]. To evaluate service quality, customers typically assess five dimensions: reliability, responsiveness, assurance, empathy, and tangibles. Reliability involves the consistent and accurate delivery of the promised service. Responsiveness pertains to the speed and willingness to assist customers. Assurance relates to employee knowledge, courteousness, and the ability to inspire trust and confidence. Empathy involves providing personalized and attentive care to customers, while tangibles refer to the physical evidence, appearance of facilities, personnel, and communication materials [65]. According to Groth et al. [14], emotional labor is associated with three of these dimensions, namely reliability, responsiveness, and assurance, as these require extensive face-to-face interactions with customers. For instance, genuine expression and deep acting by employees are more likely to have a positive impact on customers’ perception of service quality, as they are considered authentic (improving reliability) and increase customer confidence (enhancing assurance). Additionally, authenticity may lead customers to believe that employees are genuinely willing to help (increasing responsiveness). Conversely, surface acting, which is associated with a lack of authenticity, can make customers question the reliability and responsiveness of employees, thereby reducing their confidence in the service firm and ultimately diminishing service quality. Therefore, we propose:

**Proposition** **15.** *There is expected to be a negative relationship between surface acting and perceived service quality*.

**Proposition** **16.** *There is expected to be a positive relationship between deep acting and perceived service quality*.

**Proposition** **17.** *There is expected to be a positive relationship between genuine expression and perceived service quality.*

#### 3.3.3. Customer Satisfaction, Perceived Service Quality, and Customer Retention

Marketers have come to the realization that keeping existing customers is more financially efficient and advantageous than acquiring new ones [7,65]. Therefore, contemporary marketing trends have focused on implementing various marketing strategies to retain customers. Among various antecedents, customer satisfaction has been found to lead to customer retention, which, in turn, generates more revenues and market shares [63]. In fact, high satisfaction and intention ratings are often considered as loyalty by practitioners. Numerous studies have shown that customer satisfaction translates into positive organizational outcomes through the mediation of customer loyalty [62,65,70,71] and customer engagement [72]. Additionally, previous literature in service marketing has identified perceived service quality as a key contributor to customer retention [8,73,74,75].

**Proposition** **18.** *Customer satisfaction is expected to increase customer retention*.

**Proposition** **19.** *Perceived service quality is expected to increase customer retention*.

### 3.4. Moderator of Interpersonal Consequences

#### 3.4.1. Detection Ability

Social psychology researchers argue that although employees can hide their true emotions from customers, customers have the ability to detect employees’ true feelings depending on their accuracy in recognizing emotions. This is because employees sometimes unconsciously leak their true emotions [22]. Thus, the effect of emotional labor techniques on organizational results may differ depending on the customers’ accuracy in recognizing emotions. When employees display authentic emotions but customers do not recognize them, the benefits of deep acting are reduced. Similarly, if customers accurately perceive surface acting by employees, the negative impact on customer outcomes will be more significant. However, if surface acting goes unnoticed by customers, it may not have a significant effect on customer outcomes. Therefore, the connection between emotional labor techniques and customer outcomes can be distinguished based on the customers’ ability to recognize emotions.

**Proposition** **20.** *Customer detection of surface acting may affect its relationship with organizational outcomes*.

**Proposition** **21.** *Customer detection of deep acting and genuine expression may affect their relationships with organizational outcomes*.

#### 3.4.2. Relationship Strength

The term “relationship strength” refers to the frequency and depth of interactions between an employee and a customer, as well as the rapport they share [76]. Strong relationships between employees and customers are more likely to persist and withstand challenges, while weaker relationships are more prone to breaking down [42]. According to Wang and Groth’s [42] research, customers who have strong service relationships may be less inclined to process emotional information that conflicts with their existing beliefs about the employee. This is in line with the concept of confirmation bias, which states that individuals tend to favor information that supports their pre-existing beliefs. In service relationships, customers may develop a high level of trust, emotional attachment, and sometimes even friendship with the employee [77]. Due to the potential ambiguity and error associated with cues linked to inauthentic emotional displays [14], customers who have established strong relationships with employees may interpret emotional information in a manner that does not pose a threat to their relationship, even if the display is not authentic. This may result in them disregarding or overlooking indications of inauthentic emotional displays. 

In contrast, customers in weak relationships with service providers tend to rely more on emotional information displayed during service delivery, making them more likely to perceive inauthentic displays, which could result in lower satisfaction. Customers in weak relationships are not influenced by pre-existing beliefs and may interpret emotional information differently than customers in strong relationships. As a result, the impact of surface acting on customer satisfaction may be weaker for customers in strong relationships compared to those in weak relationships. Wang and Groth [42] discovered that when relationship strength is low, suppressing negative emotions through surface acting had a negative association with customer satisfaction.

**Proposition** **22.** *The relationship strength may moderate the impact of surface acting on organizational outcomes*.

### 3.5. Antecedent of Emotional Labor

#### 3.5.1. Emotional Intelligence

Emotional intelligence is a critical factor affecting the amount and type of emotional labor an individual engages in [22,49,50,78]. It refers to the capacity to recognize, comprehend, handle, and apply emotions in oneself and others [79]. The ability model of emotional intelligence [79] views EI as a set of competencies that can be learned and improved over time. This model introduced the first formal definition of EI and conducted empirical research on the four interrelated mental processes involved in emotional information: (a) emotion appraisal, (b) emotion comprehension, (c) emotion regulation, and (d) emotion utilization.

Appraisal theories propose that individuals’ emotions are triggered by their evaluations and perceptions of their circumstances and experiences. In other words, while it may seem that situations provoke people’s emotions, appraisal theory proposes that different emotions can be evoked when situations are evaluated or appraised differently [80]. Regarding emotional labor strategies, deep acting, which requires employees’ cognitive activity to alter their inner feelings, may be related to emotional intelligence, which has been regarded as the cognitive ability associated with emotions [79]. Conversely, surface acting, which disregards the cognitive activity of the appraisal stage and deals with aroused emotions to alter outward expression, may be negatively linked to emotional intelligence [19]. Additionally, emotional intelligence enables individuals to quickly recognize their own and others’ feelings, regulate their emotions more effectively, and express appropriate emotions more easily [22]. These abilities may lead employees to engage in genuine expression strategies. Based on the above, the following propositions are put forward.

**Proposition** **23.** *Emotional intelligence is expected to be negatively related to surface acting and positively related to deep acting and genuine expression*.

#### 3.5.2. Affectivity

Affectivity refers to the sum total of individual mood states [81] and has been identified as an important individual characteristic that can influence an employee’s adoption of emotional labor strategies in previous studies. Morris and Feldman [82] have stated that an individual’s tendency to experience positive and negative affect has implications for their adoption of emotional labor strategies. Watson and colleagues [81] further classified affectivity into two categories: positive affectivity and negative affectivity, both of which were found to influence emotional labor differently. Positive affectivity refers to the inclination of an individual to experience positive emotions. This trait has been linked to successful coping strategies, such as active coping, which involves modifying the nature of the stressor or one’s perception of it. The process involving positive affectivity is comparable to the process of deep acting discussed previously [83]. As a result, it is suggested that positive affectivity is positively related to deep acting and negatively associated with surface acting, which is a more superficial emotional labor strategy. Additionally, high positive affectivity employees tend to display active, enthusiastic, and positive characteristics, allowing them to display positive emotions more naturally and automatically in most given situations, which is beneficial in participant service contexts where employees are required to display positive emotions more often [84,85]. On the other hand, negative affectivity, which is defined as an individual’s tendency to experience negative emotions, can lead to pessimistic views of oneself and the world, as well as passive reactions to negative events. Employees with high negative affectivity may use emotional labor to hide negative emotions and display positive emotions superficially, also known as surface acting, when dealing with negative events, as they may realize that they should not respond negatively (see a meta-analysis of [41,84,85]). Therefore, it is suggested that negative affectivity is positively correlated with surface acting and negatively correlated with deep acting.

**Proposition** **24.** *Positive affectivity will be negatively related to (a) surface acting and positively related to (b) deep acting and (c) genuine expression*.

**Proposition** **25.** *Negative affectivity will be positively related to (a) surface acting*.

## 4. Limitations and future research directions

Although this study presents a comprehensive conceptual framework of emotional labor in sport organizations, it is important to note that this model has been developed based on theories and empirical evidence from other scientific domains. In order to fully utilize the proposed model and its applicability to the unique context of participant sport organizations, empirical evidence from sport organizations needs to be verified. Therefore, it is imperative that future research tests this model empirically in order to better understand the dynamic nature of emotional labor in sport organizations and to enhance the generalizability of the proposed framework.

To fully understand the dynamics of emotional labor in sport organizations, it is important to employ diverse research methods beyond the traditional cross-sectional self-administered questionnaire approach. While this approach has been useful in past studies of emotional labor, recent research has utilized innovative methods to address its limitations. For example, employee–customer dyadic surveys [42] can be used to collect data from both employees and customers immediately after their interactions. Latent profile analysis, as used by Nguyen, Cheung, and Stinglhamber [86], allows researchers to identify distinct emotional labor actor profiles and examine the antecedents and consequences of these profiles. Experience sampling methodology, as used by Scott and Barnes [87], involves collecting data on emotional labor strategies and their consequences at multiple points throughout the workday. Employing these and other innovative methods can provide a more comprehensive understanding of emotional labor in sport organizations, its antecedents, and consequences.

Additionally, while this model incorporates various moderators in the associations between emotional labor strategies and outcome variables, it is important to consider the impact of individual differences, such as personality traits, on emotional labor dynamics. Some studies have found that individuals with high levels of emotional intelligence may be better equipped to handle emotional labor demands, whereas individuals with certain personality traits, such as neuroticism, may be more susceptible to negative consequences of emotional labor [22]. Therefore, future research should also examine the moderating effects of individual differences on emotional labor strategies and their consequences in sport organizations. By taking into account these various factors, a more comprehensive understanding of emotional labor in sport organizations can be achieved, which may ultimately contribute to the development of effective management strategies for improving both employee well-being and customer outcomes.

Finally, sport organizations may consider the implementation of emotional labor training workshops. These workshops can assist employees in adopting more effective and health-promoting emotional labor strategies, such as deep acting and genuine expression. These training sessions may be designed to enhance employees’ emotional intelligence, develop effective emotional regulation strategies, and promote their well-being while providing high-quality service to customers. Furthermore, sport organizations may provide ongoing support, feedback, and recognition to their employees to help them manage emotional labor challenges effectively and maintain their motivation and job satisfaction.

## 5. Conclusions

Improving customer outcomes relies heavily on the quality of interactions between employees and customers, and recent service management has recognized the importance of emotions and emotional labor in creating positive experiences during customer interactions [14]. However, research in psychology has also shown that emotional labor can negatively impact the well-being of service employees, as it can be taxing and unpleasant. Therefore, understanding the role of emotional labor in employee well-being and organizational performance is critical for effective relationship marketing strategies and human resource management. Despite the importance of emotional labor in participant sport service organizations, this topic has received little attention in sport organizations. To address this gap, this study proposes a conceptual framework of emotional labor and presents 26 propositions on emotional labor dynamics in participant sport service. The framework explains the overall concept of emotional labor, the factors that influence the types and levels of emotional labor strategies, the individual and organizational consequences resulting from different emotional labor strategies, and some potential moderators of the relationship between emotional labor strategies, individual well-being, and organizational outcomes.

## Data Availability

Not applicable.
